# Association of high obesity with PAM50 breast cancer intrinsic subtypes and gene expression

**DOI:** 10.1186/s12885-015-1263-4

**Published:** 2015-04-14

**Authors:** Marilyn L Kwan, Candyce H Kroenke, Carol Sweeney, Philip S Bernard, Erin K Weltzien, Adrienne Castillo, Rachel E Factor, Kaylynn S Maxfield, Inge J Stijleman, Lawrence H Kushi, Charles P Quesenberry, Laurel A Habel, Bette J Caan

**Affiliations:** 1 Division of Research, Kaiser Permanente Northern California, 2000 Broadway, Oakland, CA 94612 USA; 2 Department of Internal Medicine, Division of Epidemiology, University of Utah, Salt Lake City, UT USA; 3 Huntsman Cancer Institute, University of Utah, Salt Lake City, UT 84112 USA; 4 The Associated Regional and University Pathologist Institute for Clinical and Experimental Pathology, Salt Lake City, UT USA

**Keywords:** Breast cancer, Obesity, Body mass index, PAM50 intrinsic subtype classifier, Tumor subtype, Gene expression, *ESR1*, *PGR*, *ERBB2*, Proliferation

## Abstract

**Background:**

Invasive breast cancers are now commonly classified using gene expression into biologically and clinically distinct tumor subtypes. However, the role of obesity in breast tumor gene expression and intrinsic subtype is unknown.

**Methods:**

Early-stage breast cancer (BC) patients (n = 1,676) were sampled from two prospective cohorts. The PAM50 qRT-PCR assay was used to: a) assess tumor gene expression levels for *ESR1, PGR, ERBB2*, and 10 proliferation genes and b) classify tumors into intrinsic subtype (Luminal A, Luminal B, Basal-like, HER2-enriched, Normal-like). Body mass index (BMI) around BC diagnosis (kg/m^2^) was categorized as: underweight (<18.5), normal (18.5-24), overweight (25–29), mildly obese (30–34), and highly obese (≥35). In a cross-sectional analysis, we evaluated associations of BMI with gene expression using linear regression models, and associations of BMI with non-Luminal A intrinsic subtypes, compared with Luminal A subtype, using multinomial logistic regression. Statistical significance tests were two-sided.

**Results:**

Highly obese women had tumors with higher expression of proliferation genes compared with normal weight women (adjusted mean difference = 0.44; 95% CI: 0.18, 0.71), yet mildly obese (adjusted mean difference = 0.16; 95% CI: −0.06, 0.38) and overweight (adjusted mean difference = 0.18; 95% CI: −0.01, 0.36) women did not. This association was stronger in postmenopausal women (p for interaction = 0.06). Being highly obese, however, was inversely associated with *ESR1* expression (adjusted mean difference = −0.95; 95% CI: −1.47, −0.42) compared with being normal weight, whereas being mildly obese and overweight were not. In addition, women with Basal-like and Luminal B subtypes, relative to those with Luminal A subtype, were more likely to be highly obese, compared with normal-weight.

**Conclusions:**

ER expression may not increase correspondingly with increasing degree of obesity. Highly obese patients are more likely to have tumor subtypes associated with high proliferation and poorer prognosis.

**Electronic supplementary material:**

The online version of this article (doi:10.1186/s12885-015-1263-4) contains supplementary material, which is available to authorized users.

## Background

Invasive breast cancers are now commonly classified using gene expression into biologically and clinically distinct tumor subtypes known as Luminal A, Luminal B, Basal-like, and HER2-Enriched (HER2-E) [1,2]. Subtype information has been shown to be an independent predictor of breast cancer survival when used in multivariate analyses including standard clinicopathologic variables [3-6]. In 2009, Parker et al. derived a minimal gene set (PAM50) for classifying “intrinsic” subtypes of breast cancer [6,7]. The PAM50 gene set has high classification agreement with larger “intrinsic” gene sets previously used for subtyping [1,2,4,6], and is a feasible assay for application in clinical and epidemiologic studies that routinely use processed tumor tissue [8].

Substantial evidence suggests that obese women are at greater risk of postmenopausal breast cancer and have poorer breast cancer survival compared with normal-weight women [9-16]. Obesity might impact breast tumor development via increased estradiol production in adipose tissue in postmenopausal women, higher insulin levels, cellular interaction of leptin with insulin, and a constant pro-inflammatory state [17,18]. However, to our knowledge, the relationship between obesity before cancer diagnosis and likelihood of a specific tumor gene profile has not been examined. Therefore, in a cohort of 1,676 breast cancer survivors derived from two large prospective cohort studies, we explored cross-sectional associations of body mass index (BMI) around breast cancer diagnosis with PAM50-derived tumor expression of selected genes (*ESR1, PGR, ERBB2*, and proliferation) and intrinsic subtype.

## Methods

### Study population

The underlying study population was women from the LACE (PI: BJ Caan, [19]) and Pathways (PI: LH Kushi, [20]) prospective cohort studies of breast cancer survivors. A total of 2,135 LACE participants were 18–79 years old when diagnosed with early-stage breast cancer from 1997–2000 (AJCC stage I with tumor size ≥1 cm, stage II, or stage IIIA) and were identified primarily from the KPNC Cancer Registry (83%) or the Utah Cancer Registry (12%). Additional eligibility criteria included: being within 39 months of diagnosis to study enrollment (mean time = 23 months, 61% between 12 and 24 months), completion of chemotherapy or radiotherapy, no prior history of breast cancer or other cancer in the last 5 years.

The Pathways Study enrolled 4,505 women diagnosed with AJCC Stage I-IV breast cancer from 2006–2013 at KPNC with no previous diagnosis of other invasive cancer, at least 21 years of age at diagnosis, and spoke English, Spanish, or Chinese. Most women were approached for enrollment within two months of diagnosis (mean time = 1.8 months, range = 0.3-7.2 months).

Participants provided informed consent under human subjects’ protocols approved by the institutional review boards (IRB) at KPNC (CN-98BCaan-04-H) and the University of Utah (IRB_00038002). All human subjects’ research carried out in this study was in compliance with the Helsinki declaration (http://www.wma.net/en/30publications/10policies/b3/index.html).

### Clinicopathologic characteristics

Clinicopathologic characteristics at cancer diagnosis, including disease stage, tumor size, nodal status, grade, estrogen receptor (ER) status, progesterone receptor (PR) status, and human epidermal growth factor receptor 2 (Her2) overexpression or amplification in the primary tumor, were abstracted from cancer registry data and medical record review.

### Obesity and other covariates

Demographic and breast cancer risk factor data were collected at study enrollment on a mailed questionnaire (LACE) or in-person interview (Pathways),and included age at breast cancer diagnosis, race/ethnicity, education, menopausal status, smoking, and moderate-vigorous physical activity (metabolic equivalent (MET)-hours/week).

LACE women were asked to self-report their weight and height at 12 months before breast cancer diagnosis on the baseline questionnaire, which as completed on average 2 years post-diagnosis. Pathways women were asked to self-report their weight and height at the time of the baseline interview, conducted on average 2 months after breast cancer diagnosis. Then a BMI value which represents the period around breast cancer diagnosis was computed from these self-reported weight and height values, and categorized according to WHO international guidelines as a 5-level variable based on our previous work in obesity and breast cancer survival [21,22]. These categories (kg/m^2^) are: underweight (<18.5), normal (18.5-24), overweight (25–29), mildly obese (30–34), and highly obese (≥35).

### Sampling strategy for PAM50 assay

For the PAM50 ancillary study (PI: BJ Caan), the LACE and Pathways cohorts were pooled with the overall goal to evaluate the performance of the PAM50 assay in a population-based study where patient characteristics, treatment patterns, and time of initial follow-up varied [23]. All LACE women from KPNC and Utah were eligible for the sub-study (n = 2,135), whereas Pathways women diagnosed from 2006–2008 were eligible (n = 2,172).

To further select eligible women for the PAM50 assay given limited study resources, we used a stratified case-cohort study design [24], with strata defined by clinical subtype based on immunohistochemistry (IHC) results for ER, PR, and Her2 [25]. The subcohort consisted of a random sample of women with the most common IHC subtype (ER+ or PR+, Her2-) (sampling fraction = 18%), and all women with the remaining less common subtypes having worse prognosis (ER+ or PR+, Her2+; ER-, PR-, Her2-; and ER-, PR-, Her2+) (sampling fraction = 100%). The cohort was followed for recurrence and survival through August 2013. Women who were not part of the subcohort but had an outcome of interest during this time were included. Out of 2,087 women selected for the case-cohort, 1,691 had tumor tissue successfully assayed by the PAM50. For this analysis, an additional 15 women were excluded due to missing BMI values, thus a total of n = 1,676 women comprised the final analytic sample with PAM50 data.

### Tissue samples

For those selected into the case-cohort study, we obtained formalin-fixed, paraffin-embedded (FFPE) tissue blocks and corresponding slides from the surgical hospital or pathology storage facility. Slides were reviewed by one pathologist (R.E.F.). If the area of invasive tumor was observed to be smaller than 0.5 cm in diameter, the case was classified as ineligible. Tissue punches 1 mm in diameter were obtained from an area of the FFPE tissue block corresponding to the marked slide.

### Gene expression assay and PAM50 intrinsic subtypes

Real-time reverse-transcription PCR (qRT-PCR) was conducted for the 50 target genes that comprise the PAM50 intrinsic subtype classifier [6], and details have been provided elsewhere [26,27]. Laboratory personnel (I.J.S.) were blinded to clinical information and received only a study identification number to track the sample.

The PAM50 assay yields an expression value for each gene that is relative to a reference gene. The raw data include both positive and negative values. For ease of interpretation of summary statistics, we transformed the values by adding 10 to all scores prior to analysis to make all values positive while preserving rank order. To determine intrinsic subtypes from the gene expression data, we applied centroid-based algorithms to the calibrated log-expression ratio for the 50 genes in the entire PAM50 assay. For each sample, this process generates five continuous-scale normalized subtype scores representing degree of Spearman correlation of gene expression with that of prototype Luminal A, Luminal B, Basal-like, HER2-E, and Normal-like breast tumors [6,26]. The subtype with the highest score became the predicted intrinsic subtype for that case.

Quantitative expression of individual genes that are standard breast cancer prognostic biomarkers (*ESR1*, *PGR*, *ERBB2*) were selected as variables of interest. Expression of 10 cell cycle regulation genes was averaged into a cell proliferation value (*CENPF, ANLN, CDC20, CCNB1, CEP55, MYBL2, MKI67, UBE2C, RRM2, KIF2C*).

### Statistical analysis

All analyses incorporated sampling weights and the stratified sampling design for unbiased estimation of population parameters and valid estimates of standard errors [28,29]. This includes estimates of frequency distributions and chi-square tests of baseline characteristics and regression analyses using the ‘svy’ commands in Stata software, StataCorp, College Station, TX. Statistical significance tests were two-sided.

We described associations of obesity with intrinsic subtypes by fitting a multinomial logistic regression model. This method is similar to the case-case analysis approach widely used for dichotomous tumor characteristics [30], extended to the five subtype categories via the multinomial model. Treating the most prevalent subtype, Luminal A, as the base comparator outcome, we estimated odds ratios (OR) and 95% confidence intervals (CI) associated with BMI categories for each of the non-Luminal A subtypes. We also used multiple linear regression for point and interval estimation of adjusted differences in mean gene expression levels across BMI categories.

All models were adjusted for age at diagnosis, race/ethnicity, moderate-vigorous physical activity, and AJCC stage. Given that associations between obesity and breast cancer risk differ by menopausal status [31], models were also stratified by menopausal status (premenopausal vs. postmenopausal). In the non-stratified models, effect modification was evaluated by calculating p values for interaction via cross-product terms of BMI as a continuous variable and menopausal status.

All models were also run individually by cohort, and results were largely consistent with the combined cohort (Additional files 1 and 2), thus we present findings for the combined cohort below.

## Results

The distributions of demographic, clinical, and PAM50 intrinsic subtype by BMI are given in Table 1. Among the highly obese (≥35 kg/m^2^) and underweight (<18.5 kg/m^2^) groups, Basal-like subtypes were more common (18.9% and 22.6%, respectively), compared with the other BMI groups (8%-10%), whereas Luminal A was less common (36.3% and 33.9%, respectively), compared with the other groups (49%-56%). African American women were more likely to be highly obese (14.9%) and less likely to be normal weight, overweight, or mildly obese (3.7%-8.7%), whereas White women were more likely to be normal weight, overweight, or mildly obese (74.2%-75.1%) and less likely to be highly obese (59.4%). Both the highly obese and underweight groups had noticeably lower levels of moderate-vigorous physical activity (67.6% and 66.7% below median 18.9 MET-hours/week, respectively), in contrast to the other BMI groups that had higher (normal weight) or similar (overweight and mildly obese) levels relative to the median level. The normal-weight women had less comorbidities (9.1%) whereas the underweight women had more comorbidities (22.6%), compared with the other BMI groups (9.1%-18.2%).

**Table 1:** **Demographic and clinical characteristics by BMI around breast cancer diagnosis, LACE and Pathways cohorts**

		Underweight	Normal weight	Overweight	Mildly Obese	Highly Obese	Total	p value^a^
		<18.5 kg/m^2^	18.5-24.9 kg/m^2^	25.0-29.9 kg/m^2^	30-34.9 kg/m^2^	≥35.0 kg/m^2^		
		n=13	n=658	n=509	n=290	n=206	n=1,676	
**Age at breast cancer diagnosis (years)**								0.04
	<50	49.2%	24.6%	20.0%	23.1%	18.3%	22.4%	
	50-64	28.2%	48.1%	43.9%	42.2%	58.2%	46.7%	
	≥65	22.6%	27.3%	36.1%	34.7%	23.5%	30.9%	
**Race/Ethnicity**								<0.0001
	White	88.7%	74.2%	74.1%	75.1%	59.4%	72.8%	
	African American	0.0%	3.7%	5.3%	8.7%	14.9%	6.3%	
	Hispanic	0.0%	5.2%	9.8%	9.4%	18.7%	8.8%	
	Asian	11.3%	14.6%	9.5%	4.3%	2.2%	9.7%	
	Other	0.0%	2.4%	1.4%	2.5%	4.9%	2.3%	
**Menopausal status at breast cancer diagnosis**								0.37
	Postmenopausal	45.2%	67.2%	71.1%	70.7%	72.5%	69.5%	
	Premenopausal	54.8%	24.8%	22.7%	25.8%	21.9%	24.2%	
	Unknown	0.0%	8.0%	6.2%	3.6%	5.5%	6.3%	
**Education**								<0.0001
	High school or less	16.9%	15.1%	25.3%	26.7%	20.1%	20.9%	
	Some college	22.6%	34.4%	36.7%	42.2%	49.2%	38.1%	
	College graduate	49.2%	24.3%	21.8%	17.1%	14.2%	21.2%	
	Post-graduate	11.3%	26.2%	16.1%	14.0%	16.5%	19.7%	
**Smoking history at breast cancer diagnosis**								0.65
	Never	28.2%	55.3%	50.9%	53.1%	50.2%	52.9%	
	Past	66.1%	38.7%	42.7%	39.2%	46.0%	40.9%	
	Current	5.6%	6.0%	6.4%	7.7%	3.8%	6.2%	
**Moderate-vigorous physical activity before breast cancer diagnosis (median=18.9 MET-hrs/wk)**								<0.0001
	Below Median	66.7%	38.0%	51.0%	58.6%	67.6%	49.2%	
	Above Median	33.3%	62.0%	49.0%	41.4%	32.4%	50.8%	
**AJCC stage**								0.20
	I	39.5%	52.3%	50.7%	49.4%	40.5%	49.9%	
	II	54.8%	41.7%	42.8%	39.9%	49.9%	42.6%	
	III	5.6%	5.6%	5.6%	10.5%	9.1%	6.9%	
	IV	0.0%	0.4%	0.9%	0.2%	0.6%	0.5%	
**Any comorbidity (Charlson comorbidity index)**								0.01
	No	77.4%	90.9%	81.8%	86.4%	83.0%	86.4%	
	Yes	22.6%	9.1%	18.2%	13.6%	17.0%	13.6%	
**PAM50 intrinsic subtype**								0.01
	Luminal A	33.9%	55.9%	49.2%	56.2%	36.3%	51.7%	
	Luminal B	37.9%	17.8%	23.4%	18.3%	26.6%	20.7%	
	Basal-like	22.6%	8.0%	9.8%	10.4%	18.9%	10.3%	
	HER2-E	5.6%	14.5%	13.6%	11.1%	17.1%	13.8%	
	Normal	0.0%	3.7%	4.0%	4.0%	1.1%	3.6%	

Note: Percentages are weighted due to stratified case-cohort study design with strata defined as IHC clinical subtype.

^a^From Pearson chi-square tests.

*ESR1* and proliferation unadjusted gene expression levels varied by BMI category, whereas there were no significant differences for *PGR* and *ERBB2* levels (Table 2). Women who were highly obese or underweight had lower *ESR1* gene expression (highly obese mean = 11.54, underweight mean = 10.95; p = 0.03) yet higher expression of proliferation genes (highly obese mean = 9.12, underweight mean = 9.07; p = 0.02), relative to women in the other BMI categories. When stratifying by menopausal status, lower *ESR1* expression in the highly obese (mean = 10.78) and underweight (mean = 10.61) continued to be observed in premenopausal women only (p = 0.01), whereas higher proliferation expression was seen in postmenopausal women who were highly obese (mean = 9.09) but not underweight (mean = 8.40, p = 0.01).

**Table 2:** **PAM50 gene expression levels by BMI around breast cancer diagnosis and menopausal status, LACE and Pathways cohorts**

	Underweight	Normal weight	Overweight	Mildly Obese	Highly Obese	p value^a^
	<18.5 kg/m^2^	18.5-24.9 kg/m^2^	25.0-29.9 kg/m^2^	30-34.9 kg/m^2^	≥35.0 kg/m^2^	
	mean	mean	mean	mean	mean	
	n=13	n=658	n=509	n=290	n=206	
**Overall (n=1,676)**						
*ESR1*	10.95	12.31	12.32	12.43	11.54	0.03
*PGR*	6.73	7.98	7.82	8.33	7.76	0.86
*ERBB2*	13.18	13.84	13.78	13.71	13.68	0.08
Proliferation	9.07	8.64	8.75	8.79	9.12	0.02
**Premenopausal (n=428)**						
*ESR1*	10.61	11.93	11.78	11.70	10.78	0.01
*PGR*	7.57	7.74	7.51	8.44	7.64	0.79
*ERBB2*	12.84	13.76	13.96	13.87	13.42	0.32
Proliferation	9.62	9.16	9.17	9.13	9.15	0.85
**Postmenopausal (n=1,135)**						
*ESR1*	11.37	12.50	12.54	12.73	11.80	0.17
*PGR*	5.71	8.01	7.96	8.31	7.77	0.76
*ERBB2*	13.59	13.87	13.73	13.67	13.76	0.15
Proliferation	8.40	8.49	8.60	8.66	9.09	0.01

NOTE: Raw values are re-scaled by adding a constant of 10 units to interpret and preserve rank order.

^a^ P values from generalized linear model (GLM) for gene expression.

The adjusted mean differences in expression of *ESR1, PGR, ERBB2*, and proliferation genes by BMI category are given in Table 3. In models adjusted for age, race/ethnicity, moderate-vigorous physical activity, AJCC stage, and study, women who were highly obese (≥35 kg/m^2^) had tumors with 0.37 standard deviation (SD) higher expression of proliferation genes vs. normal-weight women (adjusted mean difference = 0.44; 95% CI: 0.18, 0.71), yet expression levels were fairly similar at 0.13 SD for the mildly obese (30.0-34.9 kg/m^2^) vs. normal-weight women (adjusted mean difference = 0.16; 95% CI: −0.06, 0.38). By contrast, the highly obese group had 0.34 SD lower expression of *ESR1* compared with normal-weight women (adjusted mean difference = −0.95; 95% CI: −1.47, −0.42). Furthermore, compared with normal-weight women, the underweight women not only had 0.80 SD lower *ESR1* expression (adjusted mean difference = −2.24; 95% CI: −3.81, −0.67 vs. 0), but 0.77 SD lower PGR expression as well (adjusted mean difference = −2.31; 95% CI: −3.98, −0.64). No associations of BMI with *ERBB2* expression were observed.

**Table 3:** **Adjusted mean difference in gene expression levels by BMI, overall and by menopausal status**

	Overall^a^			Premenopausal^a^			Postmenopausal^a^		
*ESR1*	Total n	Mean Diff	95% CI	Total n	Mean Diff	95% CI	Total n	Mean Diff	95% CI
**BMI (kg/m** ^**2**^ **)**									
Underweight (<18.5)	13	-2.24	-3.81, -0.67	5	-3.90	-6.44, -1.36	8	-1.45	-3.24, 0.33
Normal weight (18.5-24.9)	658	Ref		179	Ref		427	Ref	
Overweight (25.0-29.9)	509	-0.12	-0.42, 0.19	112	-0.12	-0.75, 0.52	364	-0.10	-0.47, 0.26
Mildly Obese (30.0-34.9)	290	0.03	-0.36, 0.41	82	-0.12	-0.80, 0.56	195	0.10	-0.38, 0.57
Highly Obese (≥35.0)	206	-0.95	-1.47, -0.42	50	-0.96	-1.97, 0.05	141	-0.97	-1.61, -0.32
				p for interaction=0.73					
	**Overall** ^**a**^			**Premenopausal** ^**a**^			**Postmenopausal** ^**a**^		
***PGR***	**Total n**	**Mean Diff**	**95% CI**	**Total n**	**Mean Diff**	**95% CI**	**Total n**	**Mean Diff**	**95% CI**
**BMI (kg/m** ^**2**^ **)**									
Underweight (<18.5)	13	-2.31	-3.98, -0.64	5	-2.02	-5.23, 1.20	8	-2.46	-4.29, -0.62
Normal weight (18.5-24.9)	658	Ref		179	Ref		427	Ref	
Overweight (25.0-29.9)	509	-0.20	-0.62, 0.22	112	-0.08	-0.95, 0.79	364	-0.15	-0.65, 0.35
Mildly Obese (30.0-34.9)	290	0.38	-0.11, 0.88	82	1.03	0.10, 1.96	195	0.21	-0.38, 0.81
Highly Obese (≥35.0)	206	-0.13	-0.81, 0.55	50	0.49	-0.70, 1.68	141	-0.34	-1.18, 0.50
				p for interaction=0.25					
	**Overall** ^**a**^			**Premenopausal** ^**a**^			**Postmenopausal** ^**a**^		
***ERBB2***	**Total n**	**Mean Diff**	**95% CI**	**Total n**	**Mean Diff**	**95% CI**	**Total n**	**Mean Diff**	**95% CI**
**BMI (kg/m** ^**2**^ **)**									
Underweight (<18.5)	13	-0.27	-1.14, 0.60	5	-0.35	-2.14, 1.45	8	-0.22	-1.12, 0.68
Normal weight (18.5-24.9)	658	Ref		179	Ref		427	Ref	
Overweight (25.0-29.9)	509	-0.01	-0.16, 0.18	112	0.26	-0.14, 0.65	364	-0.04	-0.24, 0.16
Mildly Obese (30.0-34.9)	290	-0.08	-0.28, 0.13	82	0.10	-0.32, 0.52	195	-0.09	-0.34, 0.16
Highly Obese (≥35.0)	206	-0.04	-0.32, 0.23	50	-0.16	-0.67, 0.35	141	0.03	-0.32, 0.37
				p for interaction=0.68					
	**Overall** ^**a**^			**Premenopausal** ^**a**^			**Postmenopausal** ^**a**^		
**Proliferation**	**Total n**	**Mean Diff**	**95% CI**	**Total n**	**Mean Diff**	**95% CI**	**Total n**	**Mean Diff**	**95% CI**
**BMI (kg/m** ^**2**^ **)**									
Underweight (<18.5)	13	0.17	-0.65, 1.00	5	0.33	-0.42, 1.09	8	0.02	-1.18, 1.21
Normal weight (18.5-24.9)	658	Ref		179	Ref		427	Ref	
Overweight (25.0-29.9)	509	0.18	-0.01, 0.36	112	0.01	-0.32, 0.34	364	0.18	-0.04, 0.40
Mildly Obese (30.0-34.9)	290	0.16	-0.06, 0.38	82	-0.10	-0.47, 0.28	195	0.17	-0.09, 0.42
Highly Obese (≥35.0)	206	0.44	0.18, 0.71	50	-0.09	-0.53, 0.36	141	0.54	0.21, 0.86
				p for interaction=0.06					

^a^From linear regression, adjusted for age at diagnosis , race/ethnicity, moderate-vigorous physical activity, AJCC tumor stage, and study.

When stratifying by menopausal status, the postmenopausal group had similar patterns of association to the overall cohort (Table 3). Highly obese women had tumors with 0.47 SD higher expression of proliferation genes compared with normal-weight women (adjusted mean difference = 0.54; 95% CI: 0.21, 0.86), yet mildly obese (adjusted mean difference = 0.17; 95% CI: −0.09, 0.42) and overweight (adjusted mean difference = 0.18; 95% CI: −0.04, 0.40) women did not (0.15 SD and 0.16 SD, respectively). In contrast, being highly obese was associated with 0.34 lower *ESR1* expression (adjusted mean difference = −0.97; 95% CI: −1.61, −0.32) compared with normal weight, whereas being mildly obese (adjusted mean difference = 0.10; 95% CI: −0.38, 0.57) and overweight (adjusted mean difference = −0.10; 95% CI: −0.47, 0.26) were not (0.03 SD and 0.03 SD, respectively). Finally, being underweight compared with normal weight was associated with 0.83 SD lower PGR expression (adjusted mean difference = −2.46; 95% CI: −4.29, −0.62). We also examined expression levels in the very highly obese (≥40 kg/m^2^) postmenopausal women. Lower *ESR1* expression and higher proliferation gene expression were observed at similar levels in both the highly obese (35–40 kg/m^2^) and very highly obese (>40 kg/m^2^) women compared with normal-weight women (data not shown).

In premenopausal women (Table 3), we also observed 0.37 SD lower, yet borderline significant, *ESR1* expression among the highly obese (adjusted mean difference = −0.96; 95% CI: −1.97, 0.05), while *ESR1* expression was 1.5 SD significantly lower among the underweight (adjusted mean difference = −3.90; 95% CI: −6.44, −1.36). There was no association between BMI and expression of proliferation genes. Effect modification of the proliferation associations by menopausal status was borderline statistically significant (p for interaction = 0.06).

The associations of BMI with intrinsic subtype are given in Table 4. In models adjusted for age, race/ethnicity, moderate-vigorous physical activity, AJCC stage, and study, breast cancer patients with Basal-like tumors had over triple the odds of being highly obese (≥35 kg/m^2^) vs. normal weight compared to those with Luminal A tumors (OR = 3.75; 95% CI: 1.97, 7.12). Women with Luminal B tumors also had increased odds of being highly obese compared to those with Luminal A tumors (OR = 2.44; 95% CI: 1.24, 4.79). There was little evidence for increased odds of being mildly obese in women with Basal-like (OR = 1.38; 95% CI: 0.81, 2.36) and Luminal B (OR = 1.14; 95% CI: 0.67, 1.95) tumors. While women with Basal-like and Luminal B tumors had increased odds of being overweight, the odds were of lower magnitude compared with being highly obese. Similarly, cases with Basal-like tumors had elevated odds of being underweight compared with those with Luminal A tumors (OR = 7.44; 95% CI: 2.03, 27.32). However, the number of underweight women was small (n = 13) and made the confidence interval wide for this association, thus limiting the interpretation of this result.

**Table 4:** **Association of BMI around breast cancer diagnosis with PAM50 intrinsic subtype, overall and by menopausal status**

	Total n	PAM50 Intrinsic Subtype - Overall^a^												
		Luminal A	Luminal B			Basal-like			HER2-E			Normal-like		
		%	%	OR	95% CI	%	OR	95% CI	%	OR	95% CI	%	OR	95% CI
**BMI (kg/m** ^**2**^ **)**														
Underweight (<18.5)	13	0.4	1.0	0.56	0.07, 4.79	1.2	7.44	2.03, 27.32	0.2	0.73	0.09, 5.93	0.0	Not calculable	
Normal weight (18.5-24.9)	658	43.0	34.0	Ref		30.6	Ref		41.3	Ref		41.3	Ref	
Overweight (25.0-29.9)	509	28.6	34.1	1.72	1.10, 2.71	28.8	1.71	1.13, 2.61	29.6	1.07	0.69, 1.64	33.8	1.13	0.42, 3.05
Mildly Obese (30.0-34.9)	290	20.8	17.0	1.14	0.67, 1.95	19.4	1.38	0.81, 2.36	15.3	0.72	0.41, 1.29	21.5	1.10	0.35, 3.51
Highly Obese (≥35.0)	206	7.6	13.9	2.44	1.24, 4.79	20.0	3.75	1.97, 7.12	13.5	1.97	0.99, 3.89	3.4	0.45	0.11, 1.85
	**Total n**	**PAM50 Intrinsic Subtype - Premenopausal** ^**a,b**^												
		**Luminal A**	**Luminal B**			**Basal-like**			**HER2-E**			**Normal-like**		
		**%**	**%**	**OR**	**95% CI**	**%**	**OR**	**95% CI**	**%**	**OR**	**95% CI**	**%**	**OR**	**95% CI**
**BMI (kg/m** ^**2**^ **)**														
Underweight (<18.5)	5	0.3	2.7	Not calculable		1.6	9.70	1.22, 77.39	1.0	3.83	0.36, 41.95	0.0	Not calculable	
Normal weight (18.5-24.9)	179	41.3	40.6	Ref		36.3	Ref		46.9	Ref		19.9	Ref	
Overweight (25.0-29.9)	112	27.2	33.7	1.77	0.69, 4.52	27.4	1.20	0.52, 2.79	22.3	0.87	0.35, 2.16	26.7	2.96	0.41, 21.51
Mildly Obese (30.0-34.9)	82	22.5	17.0	0.85	0.31, 2.32	20.2	0.74	0.31, 1.79	14.5	0.50	0.18, 1.38	45.4	4.00	0.63, 25.42
Highly Obese (≥35.0)	50	9.0	6.0	0.97	0.21, 4.49	14.5	1.31	0.43, 4.03	15.2	1.92	0.53, 6.96	8.0	2.21	0.28, 17.67
	**Total n**	**PAM50 Intrinsic Subtype - Postmenopausal** ^**a,b**^												
		**Luminal A**	**Luminal B**			**Basal-like**			**HER2-E**			**Normal-like**		
		**%**	**%**	**OR**	**95% CI**	**%**	**OR**	**95% CI**	**%**	**OR**	**95% CI**	**%**	**OR**	**95% CI**
**BMI (kg/m** ^**2**^ **)**														
Underweight (<18.5)	8	0.4	0.2	0.82	0.10, 6.62	1.1	6.97	1.09, 44.38	0.0	Not calculable		0.0	Not calculable	
Normal weight (18.5-24.9)	427	41.9	30.9	Ref		26.8	Ref		39.4	Ref		44.1	Ref	
Overweight (25.0-29.9)	364	29.4	34.4	1.79	1.04, 3.08	29.0	1.94	1.13, 3.34	30.3	1.08	0.65, 1.82	38.6	1.20	0.39, 3.63
Mildly Obese (30.0-34.9)	195	21.6	16.5	1.07	0.56, 2.05	18.8	1.59	0.74, 3.43	16.7	0.77	0.38, 1.56	14.9	0.73	0.19, 2.75
Highly Obese (≥35.0)	141	7.2	18.0	3.10	1.44, 6.68	24.3	6.12	2.81, 13.35	13.6	2.01	0.90, 4.48	2.4	0.29	0.04, 1.83

^a^From multinomial logistic regression with comparison group = Luminal A, adjusted for age at diagnosis, race/ethnicity, moderate-vigorous physical activity, AJCC tumor stage, and study.

^b^p for interaction=0.52.

In stratified analyses of BMI and intrinsic subtype by menopausal status (Table 4), the postmenopausal group had patterns of association similar to the overall cohort. These same relationships were not present in the premenopausal group, although no effect modification by menopausal status was observed (p for interaction = 0.52).

## Discussion

In this cohort study of 1,676 breast cancer survivors, we found that extreme obesity around breast cancer diagnosis was positively associated with poorer prognostic gene expression profiles and tumor subtypes. Highly obese women were more likely to have tumors with greater expression of proliferation genes, and lower expression of *ESR1*, which are characteristics of the Basal-like subtype. Correspondingly, compared to women with the Luminal A intrinsic subtype, those with the Basal-like subtype and Luminal B subtype had increased odds of being highly obese (≥35 kg/m^2^) around breast cancer diagnosis. We did not find comparable associations among women who were overweight or mildly obese. The association with proliferation gene expression was observed in postmenopausal but not premenopausal women. To our knowledge, ours is the first study to examine the association of different levels of obesity with tumor gene expression.

Interestingly, we observed that a very high BMI (≥35 kg/m^2^) around breast cancer diagnosis, while strongly and positively associated with both proliferation-related gene expression and associated intrinsic subtypes (Basal-like and Luminal B), was negatively associated with estrogen-related gene expression and associated intrinsic subtypes (Luminal A). Surprisingly, the latter finding is potentially inconsistent with the notion that among postmenopausal women, obesity is generally associated with higher plasma levels of estradiol from adipose tissue [31] and greater risk of ER+ (primarily Luminal A) breast cancer [32,33], and suggests that even if higher circulating estradiol is available in postmenopausal women, the tumor itself may be less responsive to endogenous estrogen depending on level of obesity. In a study of adipose gene expression and weight loss changes in postmenopausal women, greater weight loss was associated with borderline increased *ESR1* expression (p for trend = 0.08) that could be possibly attributed to reduced adipose tissue inflammation [34]. Thus, perhaps at some threshold level of increasing obesity, tumor growth could be fueled by heightened inflammatory processes, rather than estrogen exposure, thus leading to decreased *ESR1* expression and lower likelihood of developing the associated intrinsic subtypes. Finally, while we found that the associations for subtype might also be present in the overweight but not the mildly obese group, the magnitude of association was much smaller and could be due in part to chance.

Our observation of a possible threshold effect at high BMI being associated with a proliferative tumor gene expression profile, and development of Basal-like and Luminal B tumors, is consistent with our previous work which identified highly obese (≥40 kg/m^2^) breast cancer patients being at greatest risk for poorer prognosis and survival [22,35]. While the underlying biological mechanism linking obesity to tumor etiology is unclear, there are some intriguing and plausible hypotheses. Obesity can influence cancer risk by increased production of inflammatory factors, insulin and insulin-like growth factors (IGFs), and altered adipokines, resulting in a state of low-grade chronic inflammation [36]. Higher activity of the phosphatidylinositol 3-kinase (PI3K)-Akt pathway, which primarily regulates cellular proliferation, migration, and survival [37], has been well-described in Basal-like tumors [38-40]. Thus, perhaps higher levels of insulin and IGFs in the highly obese can drive the growth of Basal-like tumors, but not Luminal tumors, through this pathway [41,42]. Furthermore, higher circulating glucose levels in the highly obese could potentially support biosynthesis of Basal-like tumors, which have been shown to be more glycolytic than other tumor subtypes [43].

While we observed some intriguing associations among the underweight, including lower expression of *ESR1* and *PGR* and being more likely to have a Basal-like tumor, we were limited by the small number of underweight women in our cohort (n = 13) to draw any definitive conclusions about this subgroup. To date, the role of underweight and tumor gene expression is largely unknown.

Strengths of this study include being the first to examine the relationship between obesity around breast cancer diagnosis and tumor gene expression, thus investigating potential molecular mechanisms of obesity on tumorigenesis. Given recent findings from large epidemiologic studies on high obesity and underweight, but not overweight or mild obesity being associated with poorer prognosis and survival [11,22,35], we were also able to examine the association of varying degrees of obesity in relation to subtype and gene expression. Finally, we used the PAM50 assay, which is a classification tool that has been shown to have better prognostic ability than surrogate IHC classification methods [23,26].

Several limitations should be noted. Weight and height were self-reported, yet substantial agreement between BMI based on self-reported, compared with measured, weight and height has been shown [44]. Also, the number of underweight women in our cohort was small (n = 13). However, we chose to keep the underweight group as a separate category, as we have previously observed elevated risks of breast cancer mortality in underweight women [22,35] and considered this analysis of obesity and gene expression exploratory. This was a cross-sectional analysis, thus causality could not be inferred between obesity around breast cancer diagnosis and tumor intrinsic subtype and gene expression. In addition, BMI reflects the relationship of weight to height and thus does not reflect between-individual variation in total adiposity [45] and metabolic risk profiles [46].

One should also consider that perhaps it is not weight at one timepoint around breast cancer diagnosis, but rather weight trajectories over the life course that may act on gene expression or tumor subtype [47]. Furthermore, compared with non-Hispanic Whites, African Americans and Hispanics are more likely to be obese [48,49], and African Americans are more likely to be diagnosed with poor prognosis subtypes [25,50,51]. Given these associations, as a sensitivity analysis, we restricted the statistical models to Whites only, and both subtype and gene expression results were essentially unchanged. Finally, LACE women were enrolled on average two years post-diagnosis, thus women with better prognosis subtypes (Luminal A) could have been more likely to survive to enrollment whereas those with poorer prognosis subtypes (Basal-like) were not. However, we found in other analyses that this potential survival bias was minimal [23], and again when we restricted the analyses by individual cohort, the results were similar (Additional files 1 and 2).

## Conclusions

Among women with breast cancer, particularly postmenopausal women, those who were highly obese, but not mildly obese, around breast cancer diagnosis were more likely to have breast tumors with greater expression of proliferation genes and lesser expression of *ESR1,* and possible increased odds of being diagnosed with Basal-like and Luminal B tumor subtypes. These findings suggest that etiology of tumor subtypes may vary by degree of pre-existing obesity of the patient and propose novel insights into molecular mechanisms linking obesity, ER expression, and proliferation to breast tumor development.

## Additional files

Additional file 1:
**LACE cohort results.**

Additional file 2:
**Pathways cohort results.**

## Abbreviations

LACE: Life After Cancer Epidemiology
*ESR1*: Estrogen receptor 1
*PGR*: Progesterone receptor
*ERBB2*: Epidermal growth factor receptor
ER: Estrogen receptor
PR: Progesterone receptor
HR: Hazard ratio
CI: Confidence interval
LumA: Luminal A
LumB: Luminal B
HER2-E: HER2-Enriched
Basal: Basal-like
IHC: Immunohistochemistry
qRT-PCR: Real-time reverse-transcription PCR
