# Supplementary material for: Association of high obesity with PAM50 breast cancer intrinsic subtypes and gene expression
Source: BMC Cancer. 2015 Apr 14;15:278. doi: 10.1186/s12885-015-1263-4 (PMC4403771; doi:10.1186/s12885-015-1263-4)
Supplement: Additional file 1: — LACE cohort results. [file 12885_2015_1263_MOESM1_ESM.docx]

| **Table 1. PAM50 gene expression by BMI around breast cancer diagnosis and menopausal status, LACE cohort** | | | | | | | | | | | | |
| --- | --- | --- | --- | --- | --- | --- | --- | --- | --- | --- | --- | --- |
|  | **Underweight** |  | **Normal weight** |  | **Overweight** |  | **Mildly Obese** |  | **Highly Obese** |  | **p value**^a^ |  |
|  | **<18.5 kg/m^2^** |  | **18.5-24.9 kg/m^2^** |  | **25.0-29.9 kg/m^2^** |  | **30-34.9 kg/m^2^** |  | **≥35.0 kg/m^2^** |  |  |  |
|  | **mean†**  **n=4** |  | **mean†**  **n=394** |  | **mean†**  **n=271** |  | **mean†**  **n=131** |  | **mean†**  **n=90** |  |  |  |
| **Overall (n=809)** |  |  |  |  |  |  |  |  |  |  |  |  |
| *ESR1* | 11.96 |  | 12.15 |  | 12.03 |  | 12.27 |  | 11.37 |  | 0.17 |  |
| *PGR* | 7.68 |  | 8.17 |  | 7.71 |  | 8.11 |  | 7.79 |  | 0.46 |  |
| *ERBB2* | 12.72 |  | 13.95 |  | 13.81 |  | 13.70 |  | 13.94 |  | 0.41 |  |
| Proliferation | 9.00 |  | 8.70 |  | 8.80 |  | 8.92 |  | 9.26 |  | **0.03** |  |
|  |  |  |  |  |  |  |  |  |  |  |  |  |
| **Premenopausal (n=190)** | |  |  |  |  |  |  |  |  |  |  |  |
| *ESR1* | 11.75 |  | 11.71 |  | 11.37 |  | 11.87 |  | 10.41 |  | 0.17 |  |
| *PGR* | 7.97 |  | 7.67 |  | 7.42 |  | 8.21 |  | 7.20 |  | 0.73 |  |
| *ERBB2* | 12.46 |  | 14.01 |  | 14.00 |  | 14.07 |  | 13.22 |  | 0.38 |  |
| Proliferation | 9.70 |  | 9.29 |  | 9.35 |  | 9.42 |  | 9.46 |  | 0.47 |  |
|  |  |  |  |  |  |  |  |  |  |  |  |  |
| **Postmenopausal (n=587)** | |  |  |  |  |  |  |  |  |  |  |  |
| *ESR1* | 12.66 |  | 12.35 |  | 12.22 |  | 12.47 |  | 11.60 |  | 0.41 |  |
| *PGR* | 6.71 |  | 8.27 |  | 7.85 |  | 8.01 |  | 7.82 |  | 0.53 |  |
| *ERBB2* | 13.59 |  | 13.95 |  | 13.81 |  | 13.65 |  | 14.15 |  | 0.68 |  |
| Proliferation | 6.65 |  | 8.59 |  | 8.64 |  | 8.75 |  | 9.18 |  | **0.10** |  |
| NOTE: Raw values are re-scaled by adding a constant of 10 units to interpret and preserve rank order | | | | | | | | | | | |  |
| ^a^ P values from generalized linear model (GLM) for gene expression | | | | | | | | | |  |  |  |

| **Table 2. Adjusted mean difference in gene expression levels by BMI, overall and by menopausal status** | | | | | | | | | | | |
| --- | --- | --- | --- | --- | --- | --- | --- | --- | --- | --- | --- |
|  | **Overall^a^** | | |  | **Premenopausal^a^** | | |  | **Postmenopausal^a^** | | |
| ***ESR1*** | **Total n** | **Mean Diff** | **95% CI** |  | **Total n** | **Mean Diff** | **95% CI** |  | **Total n** | **Mean Diff** | **95% CI** |
| **BMI (kg/m^2^)** |  |  |  |  |  |  |  |  |  |  |  |
| Underweight (<18.5) | 4 | -0.70 | -2.54, 1.14 |  | 2 | **-2.27** | **-3.41, -1.14** |  | 2 | 0.28 | -0.55, 1.12 |
| Normal weight (18.5-24.9) | 394 | Ref |  |  | 93 | Ref |  |  | 249 | Ref |  |
| Overweight (25.0-29.9) | 271 | -0.26 | -0.71, 0.18 |  | 44 | -0.44 | -1.73, 0.85 |  | 194 | -0.28 | -0.77, 0.21 |
| Mildly Obese (30.0-34.9) | 131 | -0.02 | -0.66, 0.62 |  | 34 | 0.40 | -0.67, 1.48 |  | 84 | -0.13 | -0.98, 0.73 |
| Highly Obese (≥35.0) | 90 | -0.74 | -1.53, 0.05 |  | 17 | -1.16 | -3.03, 0.71 |  | 58 | -0.68 | -1.62, 0.25 |
|  |  |  |  |  | p for interaction=0.65 | | | | | | |
|  | **Overall^a^** | | |  | **Premenopausal^a^** | | |  | **Postmenopausal^a^** | | |
| ***PGR*** | **Total n** | **Mean Diff** | **95% CI** |  | **Total n** | **Mean Diff** | **95% CI** |  | **Total n** | **Mean Diff** | **95% CI** |
| **BMI (kg/m^2^)** |  |  |  |  |  |  |  |  |  |  |  |
| Underweight (<18.5) | 4 | -2.43 | -4.97, 0.12 |  | 2 | **-4.02** | **-5.24, -2.79** |  | 2 | -1.64 | -4.89, 1.60 |
| Normal weight (18.5-24.9) | 394 | Ref |  |  | 93 | Ref |  |  | 249 | Ref |  |
| Overweight (25.0-29.9) | 271 | **-0.65** | **-1.22, -0.07** |  | 44 | -0.36 | -1.90, 1.18 |  | 194 | -0.64 | -1.32, 0.04 |
| Mildly Obese (30.0-34.9) | 131 | -0.13 | -0.86, 0.61 |  | 34 | 0.86 | -0.53, 2.25 |  | 84 | -0.47 | -1.35, 0.41 |
| Highly Obese (≥35.0) | 90 | -0.20 | -1.14, 0.75 |  | 17 | 0.39 | -1.87, 2.65 |  | 58 | -0.46 | -1.60, 0.67 |
|  |  |  |  |  | p for interaction=0.17 | | | | | | |
|  | **Overall^a^** | | |  | **Premenopausal^a^** | | |  | **Postmenopausal^a^** | | |
| ***ERBB2*** | **Total n** | **Mean Diff** | **95% CI** |  | **Total n** | **Mean Diff** | **95% CI** |  | **Total n** | **Mean Diff** | **95% CI** |
| **BMI (kg/m^2^)** |  |  |  |  |  |  |  |  |  |  |  |
| Underweight (<18.5) | 4 | -0.66 | -1.47, 0.16 |  | 2 | **-1.27** | **-1.79, -0.75** |  | 2 | -0.31 | -0.88, 0.26 |
| Normal weight (18.5-24.9) | 394 | Ref |  |  | 93 | Ref |  |  | 249 | Ref |  |
| Overweight (25.0-29.9) | 271 | -0.05 | -0.29, 0.20 |  | 44 | 0.10 | -0.45, 0.66 |  | 194 | -0.03 | -0.32, 0.25 |
| Mildly Obese (30.0-34.9) | 131 | -0.19 | -0.51, 0.13 |  | 34 | 0.06 | -0.57, 0.69 |  | 84 | -0.17 | -0.57, 0.23 |
| Highly Obese (≥35.0) | 90 | 0.18 | -0.23, 0.59 |  | 17 | -0.40 | -1.26, 0.47 |  | 58 | 0.36 | -0.16, 0.88 |
|  |  |  |  |  | p for interaction=0.44 | | | | | | |
|  | **Overall^a^** | | |  | **Premenopausal^a^** | | |  | **Postmenopausal^a^** | | |
| **Proliferation** | **Total n** | **Mean Diff** | **95% CI** |  | **Total n** | **Mean Diff** | **95% CI** |  | **Total n** | **Mean Diff** | **95% CI** |
| **BMI (kg/m^2^)** |  |  |  |  |  |  |  |  |  |  |  |
| Underweight (<18.5) | 4 | -1.17 | -2.41, 0.06 |  | 2 | 0.05 | -0.44, 0.53 |  | 2 | **-1.94** | **-2.68, -1.21** |
| Normal weight (18.5-24.9) | 394 | Ref |  |  | 93 | Ref |  |  | 249 | Ref |  |
| Overweight (25.0-29.9) | 271 | 0.16 | -0.07, 0.40 |  | 44 | 0.11 | -0.41, 0.63 |  | 194 | 0.08 | -0.21, 0.36 |
| Mildly Obese (30.0-34.9) | 131 | 0.22 | -0.12, 0.57 |  | 34 | 0.04 | -0.59, 0.67 |  | 84 | 0.15 | -0.20, 0.51 |
| Highly Obese (≥35.0) | 90 | **0.58** | **0.15, 1.02** |  | 17 | -0.08 | -0.81, 0.65 |  | 58 | **0.63** | **0.09, 1.17** |
|  |  |  |  |  | p for interaction=0.42 | | | | | | |
| ^a^ From linear regression, adjusted for age at diagnosis , race/ethnicity, moderate-vigorous physical activity, and AJCC tumor stage | | | | | | | | | | | |

| **Table 3. Association of BMI around breast cancer diagnosis with PAM50 intrinsic subtype, LACE cohort** | | | | | | | | | | | | | | |
| --- | --- | --- | --- | --- | --- | --- | --- | --- | --- | --- | --- | --- | --- | --- |
|  | **Total**  **n** | **PAM50 Intrinsic Subtype - Overall^a,b^** | | | | | | | | | | | | |
|  |  | **Luminal A** | **Luminal B** | | | **Basal-like** | | | **HER2-E** | | | **Normal-like** | | |
|  |  | **%** | **%** | **OR** | **95% CI** | **%** | **OR** | **95% CI** | **%** | **OR** | **95% CI** | **%** | **OR** | **95% CI** |
| **BMI (kg/m^2^)** |  |  |  |  |  |  |  |  |  |  |  |  |  |  |
| Underweight (<18.5) | 4 | 0.3 | 1.0 | Not calculable | | 0.6 | Not calculable | | 0.0 | Not calculable | | 0.0 | Not calculable | |
| Normal weight (18.5-24.9) | 394 | 52.3 | 36.1 | Ref |  | 32.0 | Ref |  | 52.1 | Ref |  | 25.7 | Ref |  |
| Overweight (25.0-29.9) | 271 | 26.6 | 33.6 | **2.08** | **1.08, 4.00** | 31.4 | 3.12 | 0.47, 20.97 | 27.8 | 1.15 | 0.63, 2.10 | 39.2 | 2.48 | 0.68, 8.98 |
| Mildly Obese (30.0-34.9) | 131 | 14.2 | 22.0 | **2.53** | **1.17, 5.49** | 20.2 | **2.68** | **1.49, 4.85** | 12.1 | 0.89 | 0.32, 2.45 | 31.1 | **5.25** | **1.44, 19.12** |
| Highly Obese (≥35.0) | 90 | 6.6 | 6.2 | 1.75 | 0.66, 4.67 | 15.8 | **2.76** | **1.17, 6.51** | 8.1 | 1.36 | 0.50, 3.71 | 4.0 | 1.89 | 0.35, 10.09 |
| ^a^ From multinomial logistic regression with comparison group = Luminal A, adjusted for age at diagnosis, race/ethnicity, moderate-vigorous physical activity, and AJCC tumor stage | | | | | | | | | | | | | | |
| ^b^ Due to limited sample size, models stratified by menopausal status could not be run | | | | | | | | | | | | | | |
