# Supplementary material for: Association of high obesity with PAM50 breast cancer intrinsic subtypes and gene expression
Source: BMC Cancer. 2015 Apr 14;15:278. doi: 10.1186/s12885-015-1263-4 (PMC4403771; doi:10.1186/s12885-015-1263-4)
Supplement: Additional file 2: — Pathways cohort results. [file 12885_2015_1263_MOESM2_ESM.docx]

| **Table 1. PAM50 gene expression by BMI around breast cancer diagnosis and menopausal status, Pathways cohort** | | | | | | | | | | | | |
| --- | --- | --- | --- | --- | --- | --- | --- | --- | --- | --- | --- | --- |
|  | **Underweight** |  | **Normal weight** |  | **Overweight** |  | **Mildly Obese** |  | **Highly Obese** |  | **p value**^a^ |  |
|  | **<18.5 kg/m^2^** |  | **18.5-24.9 kg/m^2^** |  | **25.0-29.9 kg/m^2^** |  | **30-34.9 kg/m^2^** |  | **≥35.0 kg/m^2^** |  |  |  |
|  | **mean†**  **n=9** |  | **mean†**  **n=264** |  | **mean†**  **n=238** |  | **mean†**  **n=159** |  | **mean†**  **n=116** |  |  |  |
| **Overall (n=786)** |  |  |  |  |  |  |  |  |  |  |  |  |
| *ESR1* | 9.97 |  | 12.48 |  | 12.55 |  | 12.50 |  | 11.29 |  | **0.004** |  |
| *PGR* | 5.81 |  | 7.74 |  | 7.91 |  | 8.41 |  | 7.47 |  | 0.93 |  |
| *ERBB2* | 13.62 |  | 13.72 |  | 13.77 |  | 13.70 |  | 13.55 |  | 0.24 |  |
| Proliferation | 9.14 |  | 8.59 |  | 8.74 |  | 8.73 |  | 9.05 |  | 0.13 |  |
|  |  |  |  |  |  |  |  |  |  |  |  |  |
| **Premenopausal (n=238)** | |  |  |  |  |  |  |  |  |  |  |  |
| *ESR1* | 8.05 |  | 12.08 |  | 11.94 |  | 11.60 |  | 10.87 |  | **0.03** |  |
| *PGR* | 6.67 |  | 7.74 |  | 7.56 |  | 8.56 |  | 7.76 |  | 0.68 |  |
| *ERBB2* | 13.67 |  | 13.55 |  | 13.93 |  | 13.77 |  | 13.47 |  | 0.68 |  |
| Proliferation | 9.45 |  | 9.09 |  | 9.11 |  | 8.97 |  | 9.07 |  | 0.75 |  |
|  |  |  |  |  |  |  |  |  |  |  |  |  |
| **Postmenopausal (n=548)** | |  |  |  |  |  |  |  |  |  |  |  |
| *ESR1* | 10.94 |  | 12.66 |  | 12.79 |  | 12.87 |  | 11.45 |  | **0.03** |  |
| *PGR* | 5.37 |  | 7.74 |  | 8.04 |  | 8.35 |  | 7.34 |  | 0.71 |  |
| *ERBB2* | 13.59 |  | 13.80 |  | 13.71 |  | 13.68 |  | 13.58 |  | 0.24 |  |
| Proliferation | 8.98 |  | 8.39 |  | 8.60 |  | 8.63 |  | 9.04 |  | **0.04** |  |
| NOTE: Raw values are re-scaled by adding a constant of 10 units to interpret and preserve rank order | | | | | | | | | | | |  |
| ^a^ P values from generalized linear model (GLM) for gene expression | | | | | | | | | |  |  |  |

| **Table 2. Adjusted mean difference in gene expression levels by BMI, overall and by menopausal status** | | | | | | | | | | | |
| --- | --- | --- | --- | --- | --- | --- | --- | --- | --- | --- | --- |
|  | **Overall^a^** | | |  | **Premenopausal^a^** | | |  | **Postmenopausal^a^** | | |
| ***ESR1*** | **Total n** | **Mean Diff** | **95% CI** |  | **Total n** | **Mean Diff** | **95% CI** |  | **Total n** | **Mean Diff** | **95% CI** |
| **BMI (kg/m^2^)** |  |  |  |  |  |  |  |  |  |  |  |
| Underweight (<18.5) | 9 | **-2.72** | **-4.61, -0.83** |  | 3 | **-4.22** | **-7.46, -0.97** |  | 6 | -1.97 | -4.14, 0.21 |
| Normal weight (18.5-24.9) | 264 | Ref |  |  | 86 | Ref |  |  | 178 | Ref |  |
| Overweight (25.0-29.9) | 238 | -0.00 | -0.43, 0.42 |  | 68 | -0.02 | -0.74, 0.69 |  | 170 | 0.05 | -0.47, 0.58 |
| Mildly Obese (30.0-34.9) | 159 | 0.03 | -0.45, 0.52 |  | 48 | -0.37 | -1.28, 0.54 |  | 111 | 0.21 | -0.37, 0.78 |
| Highly Obese (≥35.0) | 116 | **-1.05** | **-1.74, -0.35** |  | 33 | -0.99 | -2.17, 0.18 |  | 83 | **-1.03** | **-1.89, -0.17** |
|  |  |  |  |  | p for interaction=0.53 | | | | | | |
|  | **Overall^a^** | | |  | **Premenopausal^a^** | | |  | **Postmenopausal^a^** | | |
| ***PGR*** | **Total n** | **Mean Diff** | **95% CI** |  | **Total n** | **Mean Diff** | **95% CI** |  | **Total n** | **Mean Diff** | **95% CI** |
| **BMI (kg/m^2^)** |  |  |  |  |  |  |  |  |  |  |  |
| Underweight (<18.5) | 9 | **-2.08** | **-4.14, -0.02** |  | 3 | -1.17 | -5.00, 2.65 |  | 6 | **-2.56** | **-4.74, -0.39** |
| Normal weight (18.5-24.9) | 264 | Ref |  |  | 86 | Ref |  |  | 178 | Ref |  |
| Overweight (25.0-29.9) | 238 | 0.16 | -0.43, 0.76 |  | 68 | 0.07 | -1.01, 1.15 |  | 170 | 0.25 | -0.47, 0.98 |
| Mildly Obese (30.0-34.9) | 159 | **0.76** | **0.10, 1.43** |  | 48 | 1.16 | -0.10, 2.43 |  | 111 | 0.62 | -0.16, 1.41 |
| Highly Obese (≥35.0) | 116 | 0.01 | -0.90, 0.92 |  | 33 | 0.55 | -0.86, 1.96 |  | 83 | -0.13 | -1.23, 0.97 |
|  |  |  |  |  | p for interaction=0.74 | | | | | | |
|  | **Overall^a^** | | |  | **Premenopausal^a^** | | |  | **Postmenopausal^a^** | | |
| ***ERBB2*** | **Total n** | **Mean Diff** | **95% CI** |  | **Total n** | **Mean Diff** | **95% CI** |  | **Total n** | **Mean Diff** | **95% CI** |
| **BMI (kg/m^2^)** |  |  |  |  |  |  |  |  |  |  |  |
| Underweight (<18.5) | 9 | **-0.11** | **-1.22, 1.00** |  | 3 | 0.096 | -2.18, 2.38 |  | 6 | **-0.19** | **-1.38, 1.00** |
| Normal weight (18.5-24.9) | 264 | Ref |  |  | 86 | Ref |  |  | 178 | Ref |  |
| Overweight (25.0-29.9) | 238 | 0.06 | -0.18, 0.30 |  | 68 | 0.4 | -0.12, 0.91 |  | 170 | -0.05 | -0.32, 0.22 |
| Mildly Obese (30.0-34.9) | 159 | 0.01 | -0.26, 0.28 |  | 48 | 0.18 | -0.37, 0.74 |  | 111 | -0.05 | -0.36, 0.26 |
| Highly Obese (≥35.0) | 116 | -0.13 | -0.48, 0.23 |  | 33 | -0.054 | -0.69, 0.58 |  | 83 | -0.13 | -0.57, 0.31 |
|  |  |  |  |  | p for interaction=0.35 | | | | | | |
|  | **Overall^a^** | | |  | **Premenopausal^a^** | | |  | **Postmenopausal^a^** | | |
| **Proliferation** | **Total n** | **Mean Diff** | **95% CI** |  | **Total n** | **Mean Diff** | **95% CI** |  | **Total n** | **Mean Diff** | **95% CI** |
| **BMI (kg/m^2^)** |  |  |  |  |  |  |  |  |  |  |  |
| Underweight (<18.5) | 9 | 0.59 | -0.23, 1.41 |  | 3 | 0.31 | -0.66, 1.28 |  | 6 | 0.7 | -0.46, 1.86 |
| Normal weight (18.5-24.9) | 264 | Ref |  |  | 86 | Ref |  |  | 178 | Ref |  |
| Overweight (25.0-29.9) | 238 | 0.19 | -0.08, 0.45 |  | 68 | -0.021 | -0.42, 0.38 |  | 170 | 0.26 | -0.06, 0.59 |
| Mildly Obese (30.0-34.9) | 159 | 0.11 | -0.18, 0.40 |  | 48 | -0.2 | -0.67, 0.26 |  | 111 | 0.2 | -0.15, 0.55 |
| Highly Obese (≥35) | 116 | **0.36** | **0.03, 0.69** |  | 33 | -0.09 | -0.64, 0.46 |  | 83 | **0.5** | **0.09, 0.90** |
|  |  |  |  |  | **p for interaction=0.09** | | | | | | |
| ^a^ From linear regression, adjusted for age at diagnosis , race/ethnicity, moderate-vigorous physical activity, and AJCC tumor stage | | | | | | | | | | | |

| **Table 3. Association of BMI around breast cancer diagnosis with PAM50 intrinsic subtype, Pathways cohort** | | | | | | | | | | | | | | |
| --- | --- | --- | --- | --- | --- | --- | --- | --- | --- | --- | --- | --- | --- | --- |
|  | **Total**  **n** | **PAM50 Intrinsic Subtype - Overall^a,b^** | | | | | | | | | | | | |
|  |  | **Luminal A** | **Luminal B** | | | **Basal-like** | | | **HER2-E** | | | **Normal-like** | | |
|  |  | **%** | **%** | **OR** | **95% CI** | **%** | **OR** | **95% CI** | **%** | **OR** | **95% CI** | **%** | **OR** | **95% CI** |
| **BMI (kg/m^2^)** |  |  |  |  |  |  |  |  |  |  |  |  |  |  |
| Underweight (<18.5) | 9 | 0.5 | 0.3 | 0.71 | 0.08, 6.51 | 1.9 | **8.42** | **1.70, 41.67** | 0.4 | 1.15 | 0.13, 10.12 | 0.0 | Not calculable | |
| Normal weight (18.5-24.9) | 264 | 36.6 | 32.9 | Ref |  | 29.1 | Ref |  | 33.4 | Ref |  | 52.7 | Ref |  |
| Overweight (25.0-29.9) | 238 | 30.5 | 36.1 | 1.47 | 0.79, 2.74 | 26.0 | 1.09 | 0.59, 2.01 | 30.5 | 1.06 | 0.57, 1.99 | 29.9 | 0.67 | 0.17, 2.61 |
| Mildly Obese (30.0-34.9) | 159 | 25.1 | 14.2 | 0.62 | 0.30, 1.27 | 18.6 | 0.79 | 0.40, 1.55 | 16.8 | 0.65 | 0.31, 1.38 | 14.4 | 0.37 | 0.07, 2.07 |
| Highly Obese (≥35.0) | 116 | 7.4 | 16.5 | **2.54** | **1.05, 6.18** | 24.6 | **2.98** | **1.30, 6.79** | 18.8 | 2.36 | 0.94, 5.93 | 3.0 | 0.19 | 0.03, 1.26 |
| ^a^ From multinomial logistic regression with comparison group = Luminal A, adjusted for age at diagnosis, race/ethnicity, moderate-vigorous physical activity, and AJCC tumor stage | | | | | | | | | | | | | | |
| ^b^ Due to limited sample size, models stratified by menopausal status could not be run. | | | | | | | | | | | | | | |
